# Statins Improve Clinical Outcome After Non-aneurysmal Subarachnoid Hemorrhage: A Translational Insight From a Systematic Review of Experimental Studies

**DOI:** 10.3389/fneur.2021.620096

**Published:** 2021-05-14

**Authors:** Sepide Kashefiolasl, Marlies Wagner, Nina Brawanski, Volker Seifert, Stefan Wanderer, Lukas Andereggen, Juergen Konczalla

**Affiliations:** ^1^Department of Neurosurgery, University Hospital Frankfurt, Frankfurt am Main, Germany; ^2^Institute of Neuroradiology, University Hospital Frankfurt, Frankfurt am Main, Germany; ^3^Department of Neurosurgery, Kantonsspital Aarau, Aarau, Switzerland; ^4^Cerebrovascular Research Group, Department for BioMedical Research, University of Bern, Bern, Switzerland

**Keywords:** non-aneurysmal SAH, stroke, regenerative medicine, recovery, translational study, statin treatment

## Abstract

The efficacy of statin-treatment in aneurysmal subarachnoid hemorrhage (SAH) remains controversial. We aimed to investigate the effects of statin-treatment in non-aneurysmal (na)SAH in accordance with animal research data illustrating the pathophysiology of naSAH. We systematically searched PubMed using PRISMA-guidelines and selected experimental studies assessing the statin-effect on SAH. Detecting the accordance of the applied experimental models with the pathophysiology of naSAH, we analyzed our institutional database of naSAH patients between 1999 and 2018, regarding the effect of statin treatment in these patients and creating a translational concept. Patient characteristics such as statin-treatment (simvastatin 40 mg/d), the occurrence of cerebral vasospasm (CVS), delayed infarction (DI), delayed cerebral ischemia (DCI), and clinical outcome were recorded. In our systematic review of experimental studies, we found 13 studies among 18 titles using blood-injection-animal-models to assess the statin-effect in accordance with the pathophysiology of naSAH. All selected studies differ on study-setting concerning drug-administration, evaluation methods, and neurological tests. Patients from the Back to Bedside project, including 293 naSAH-patients and 51 patients with simvastatin-treatment, were recruited for this analysis. Patients under treatment were affected by a significantly lower risk of CVS (*p* < 0.01; OR 3.7), DI (*p* < 0.05; OR 2.6), and DCI (*p* < 0.05; OR 3). Furthermore, there was a significant association between simvastatin-treatment and favorable-outcome (*p* < 0.05; OR 3). However, dividing patients with statin-treatment in pre-SAH (*n* = 31) and post-SAH (*n* = 20) treatment groups, we only detected a tenuously significant higher chance for a favorable outcome (*p* < 0.05; OR 0.05) in the small group of 20 patients with statin post-SAH treatment. Using a multivariate-analysis, we detected female gender (55%; *p* < 0.001; OR 4.9), Hunt&Hess ≤III at admission (*p* < 0.002; OR 4), no anticoagulant-therapy (*p* < 0.0001; OR 0.16), and statin-treatment (*p* < 0.0001; OR 24.2) as the main factors improving the clinical outcome. In conclusion, we detected a significantly lower risk for CVS, DCI, and DI in naSAH patients under statin treatment. Additionally, a significant association between statin treatment and favorable outcome 6 months after naSAH onset could be confirmed. Nevertheless, unified animal experiments should be considered to create the basis for developing new therapeutic schemes.

## Introduction

Early brain injury (EBI) followed by subarachnoid hemorrhage (SAH) is caused by transient cerebral ischemia during bleeding. In up to 30% of SAH patients, delayed cerebral ischemia (DCI) is associated with poor clinical outcome or death. DCI is the result of SAH caused secondary effects such as increased intracranial pressure and destruction of brain tissue by intracerebral hemorrhage. A combination of post-SAH complications including cerebral vasospasm, arteriolar constriction and thrombosis, cortical spreading ischemia, and processes triggered by EBI lead to DCI (1–3). Therefore, numerous pharmacological treatments have concentrated on the prevention of EBI and cerebral vasospasm, with disappointing results as of yet (4).

Statins have been identified as a potential treatment option for early brain injury and the associated vasospasm. Based on experimental studies using animal models proving vascular regenerative effects of statins, statin-treatment might reduce EBI and vasospasm in patients after aneurysmal SAH (5, 6).

However, the results of clinical trials have been controversial. A decreased incidence of vasospasm was found in a few Phase II trials, while further clinical studies have failed to confirm these findings (7–9). Additionally, Meta-analyses have also produced inconsistent results. In contrast to clinical studies demonstrating a beneficial effect of statin treatment in only one aspect (10–12), there are additional studies disproving all reported benefits (13–15). Nevertheless, two large-scale multicenter Phase III trials- Simvastatin in Aneurysmal Subarachnoid Hemorrhage (STASH) (16) and High-Dose Simvastatin for Aneurysmal Subarachnoid Hemorrhage (HDS-SAH) (17)—could not detect any beneficial effect of simvastatin treatment independent of statin dose, which has been described as effective in higher drug doses, previously. Therefore, the effect of statins on vasospasm remains unclear.

Considering this discrepancy between experimental and clinical data, we aimed to investigate the effects of statin treatment in SAH in accordance with animal research data. Firstly, a systematic review was conducted to explore the research background of the role of statins in the case of SAH. Detecting the accordance of the applied experimental models with the pathophysiology of naSAH, we analyzed our patient database regarding the effect of statin treatment in non-aneurysmal SAH patients.

## Methods

### Systematic Review of Experimental Data

Peer-reviewed articles reporting the clinical evidence of efficacy of statins for SAH in experimental animal models were identified. Papers were selected in MEDLINE/Pubmed according to the PRISM statement. The search terms “statin AND SAH” with the filter option for “species” were used to search Pubmed systematically. English language articles up to December 2018 were considered. The literature review wasperformed by two independent reviewers.

All studies working on an experimental SAH model *in vivo* were included after first review of titles and abstracts. Differentiating between a blood injection and endovascular perforation SAH model (referring to the study design single or double hemorrhage model in case of both methods), animal studies with injection models were included. Blood injection models with the presence of subarachnoid blood in the cisterns around the midbrain mimicking naSAH with the center of the bleeding immediately anterior to the midbrain, with or without extension to the ambient and chiasmatic cisterns, horizontal part of the sylvian fissure and posterior horns of the lateral ventricles, resembling non-aneurysmal SAH in humans, were added to our database.

All included papers were reviewed in full text and availability of coordinates. Reference lists of suitable studies were scrutinized for additional articles.

All eligible studies were analyzed describing the following parameters: animal species, study design containing the exact description of SAH model, size of animal groups, dose of drug, route and time of drugs' administration, employed tests, time of examination, and results.

### Clinical Data

Regarding the highest number of studies using the SAH blood injection model, in accordance with the pathophysiology of naSAH, we retrospectively analyzed our institutional database of consecutive patients suffering naSAH between 1999 and 2018. Non-aneurysmal Subarachnoid hemorrhage was defined as a spontaneous non-traumatic hemorrhage into the subarachnoid space without any evidence of an intracranial vascular pathology.

The retrospective clinical study was approved by the local ethics committee of the Goethe-University and was performed in accordance with the relevant guidelines and regulations of the regional ethics committee in Frankfurt am Main, Germany. Because of the retrospective design, informed consent was waived by the ethics committee.

All patients with SAH, diagnosed by SAH pattern on CT-scan, or confirmed by lumbar puncture, underwent cerebral digital subtraction angiography (DSA) to rule out intracranial vascular bleeding sources. Patients in whom the bleeding source was detected to be an aneurysm or vascular malformation were excluded from the study and treated by surgical or endovascular aneurysm occlusion based on an interdisciplinary consensus. If absence of intracranial vascular pathology was confirmed by a neuroradiologist, patients received a 2nd angiography regularly ~14–48 days after the ictus. All admitted SAH patients without intracranial vascular pathology were included in this study as non-aneurysmal SAH patients.

According to bleeding patterns, naSAH patients were divided into perimesencephalic- (PM-) and non-perimesencephalic (NPM-) naSAH. A PM-SAH was defined as the presence of subarachnoid blood in the cisterns around the midbrain with the center of the bleeding immediately anterior to the midbrain, with or without extension to the ambient and chiasmatic cisterns, the horizontal part of the sylvian fissure, and posterior horns of the lateral ventricles. Extensive intraventricular hemorrhage and intraparenchymal hemorrhage were exclusive of PM-SAH. Patients with an aneurysmal SAH pattern, described as blood located in the interpeduncular cistern, as well as in the Sylvian cistern, interhemispheric cistern and convexity, or a negative computer tomography with a positive lumbar puncture, and negative digital subtraction angiography (DSA), were classified as NPM-SAH.

Patients not receiving treatment at SAH onset because of advanced brain injury or without clinical follow-up 6 months post-SAH were excluded.

All parameters relevant to this analysis, including statin-pretreatment (medication intake independent of SAH onset), statin-post-treatment after SAH onset (drug: simvastatin 40 mg a day), the occurrence of CVS, DI (CVS-related vs. non-CVS-related), DCI and finally clinical outcome (modified Rankin Scale: mRS after 6 months; favorable outcome mRS 0–2 vs. unfavorable outcome mRS 3–6) in patients with non-aneurysmal SAH, were recorded.

The degree of CVS in patients was uniformly defined by CT-A as a radiological imaging on the basis of arterial narrowing (e.g., >66% for severe CVS). Because of the small number of patients with high or low dose statin treatment [statin dose: 80 mg (*n* = 2) or 20 mg (*n* = 3)], these patients were excluded.

### Statistical Analysis

Data analyses were performed using the computer software package (IBM SPSS, version 22, IBM SPSS Inc.; Armonk, NY). Multivariable logistic regression analysis with forward stepwise modeling was carried out to identify factors for outcome improvement among above mentioned aspects. Categorical variables were analyzed using the Fisher's exact test and GraphPad Prism (6.0, GraphPad Software Inc., USA). Normal distributed variables were expressed as mean values with standard deviations (SD) and analyzed using a two-tailed *t-*test. A *p* < 0.05 was considered statistically significant.

## Results

### Literature Review of Experimental Data

**Figure 1:**
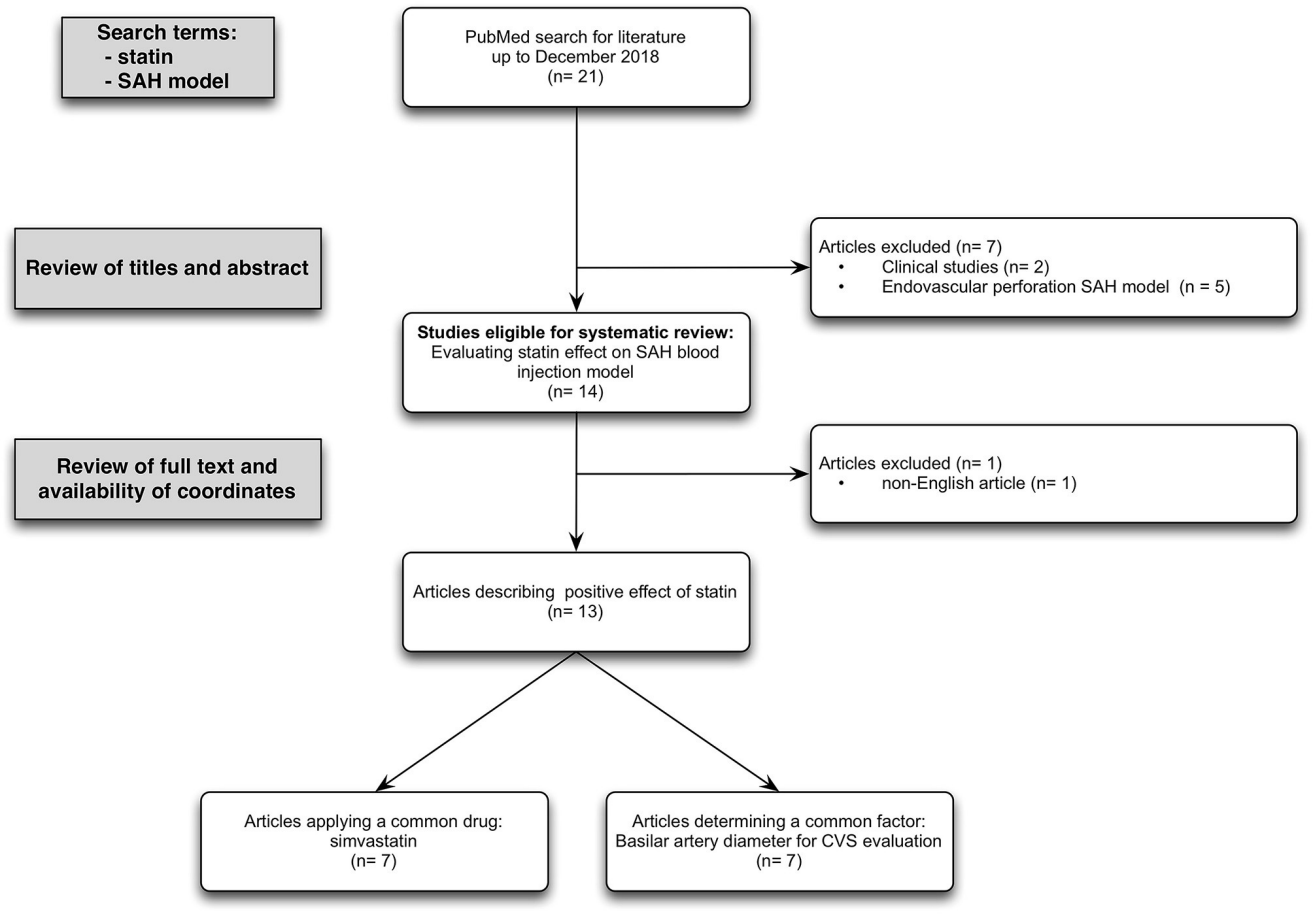
PRISMA flow diagram: Flow chart summarizing the study selection process. Inclusion criteria required animal-based experimental studies focusing on statin effect in case of SAH induced by subarachnoidal blood injection model. Exclusion criteria required studies reporting on the application of an endovascular perforation SAH model and language other than English. Overall, 13 studies met the inclusion and exclusion criterias.

Of the total of 18 English titles with experimental data, 13 studies with the presence of subarachnoid blood in the cisterns around the midbrain in accordance with the pathophysiology of naSAH used SAH blood injection model to assess the statin effect. Only seven studies applied simvastatin as the same drug, but in different doses and application routes. All of these selected studies differ in time of drug administration, employed tests, and time of examination. Evaluation of basilar artery diameter as a unified parameter to detect CVS after SAH was done in seven studies, using cerebral angiography (2/7) vs. measurement of cross-sectional areas (5/7) (Figure 1, PRISMA flow diagram).

Detailed study data summarized in Table 1 (18–29, 31) demonstrate the disunity of study setting of all included experimental studies determining the statin effect on SAH using blood injection model. There are not only discrepancies in choosing animal species, SAH model (single vs. double blood injection model) and size of animal groups, but also in study setup including choice and dose of drug, route and time of drug administration, employed tests, time of examination, and evaluated results.

**Table 1:** Experimental studies assessing neuroprotective effects of Statin on SAH using blood injection model.

**References**	**Animal model**	**Study design**	**Sample size**	**Dose**	**Route**	**Time of administration**	**Tests employed**	**Time of examination**	**Results**
McGirt et al. (18)	Rabbits	Blood injection model	*n =* 15/group	40 mg/kg/day **Simvastatin**	s.c.	30 min; 24 and 48 h after SAH/Sham operation	Measurement of Basilar Artery lumen diameter; migration of perivascular granulocytes and macrophages.	72 h after SAH	Attenuation of perivascular granulocyte migration; Amelioration of basilar artery vasospasm
Bulsara et al. (19)	Dogs	Double blood injection model	*n =* 4–5/group	20 mg/kg/day (**Simvastatin** tested with/without combination with cyclosporine)	Oral	6 h after the 2nd blood injection for 10 days	Evaluation of Basilar artery diameter by cerebral angiography	1, 3, 7, and 10 days after SAH	Simvastatin combined with cyclosporine is not as efficacious in ameliorating vasospasm as simvastatin alone
Takata et al. (20)	Rats	Double blood injection model	*n =* 12–15/group	1.5 mg/kg/day vs. 10 mg/kg/day **Simvastatin**	Oral	Low dose: after SAH for 2 and 5 weeks; High dose: after SAH for 2 weeks	Rotarod: Morris water maze test	On days 0, 1, 3, 7, 10, 14, 17, 21, 24, 28; daily on days 29–35	Low dose: improvement of neurological deficits up to 28 days; High dose: improvement of neurological deficits up to 10 days
Wang et al. (21)	Rats	Autologous blood injection model	*n =* 6/group	20 mg/kg/day **Simvastatin**	i.p.	After SAH induction	Measurement of cross-sectional areas of MCA and ACA; H&E staining evaluating micro clots	7 days after SAH	Simvastatin attenuates cerebral vasospasm and alleviates microthrombosis in the late phase of SAH
Chang et al. (22)	Rats	Double blood injection model	*n =* 9/group	5 mg/day **Atorvastartin**	Oral	Pre-condition: 1 week before SAH induction; treatment: 24 h after SAH induction	Measurement of cross-sectional areas of BA; Evaluation of vasoactive factors: ET-1 and eNOS in CSF	72 h after 2. SAH	Reduction of ET-1 level in the preconditioning status; Increase of eNOS expression in precondition and reversal groups; Increase of BA diameter in preconditioning groups
Merlo et al. (23)	Rats	Blood injection model	*n =* 12/group	40 mg/kg/days **Simvastatin**	i.p.	30 min after SAH for 5 consecutive days	Neurobehaviour testing	1–4 post-SAH day	Reduction of post-SAH cognitive dysfunction under simvastatin
Sabri et al. (24)	Mice	Prechiasmatic cistern blood injection model	*n =* 15/group	20 mg/kg/day **Simvastatin**	i.p.	Daily for 14 days before and for 2 days after SAH operation or 30 min after SAH operation and daily for 2 d	Measurement of cross-sectional areas of BA; Evaluation of vasoactive factors.	48 h post-SAH	Simvastatin can re-couple dysfunctional eNOS and restore NO/O2Γ balance, thus preventing vasospasm, microthrombo-embolism, and neuronal injury after SAH
Naraoka et al. (25)	Rabbits	Double blood injection model	*n =* 8/groups	0.8 mg/kg/day **Pitavastatin** with/without combination with 3 mg/kg 2x/day i.v. fasudil	Oral	Day 0–5 after SAH	Histological evaluation of BA diameter; Using ELISA for protein analysis (Rho-kinase, eNOS)	5 days post-SAH	Combinated treatment prevent cerebral vasospasm due to synergic effect of pitavastatin and fasudil on Rho-kinase pathway and eNOS
Platt et al. (26)	Dogs	Double blood injection model	*n =* 4–5/group	–(Unavailable)	–(Unavailable)	–(Unavailable)	Analyzing neurotransmitter concentrations in CSF	CSF was collected before 1. blood injection, before second blood injection and on days 3, 7, and 10	Simvastatin attenuated high glutamate concentrations
Chang et al. (27)	Rats	Double blood injection model	*n =* 9/group	1 mg/day **Pitavastatin**	Oral	Precondition: 1 week before SAH induction; treatment: after SAH induction	Analyzing factors inducing neuron apoptosis after SAH using qPCR	Precondition: 24 h after 1. blood injection, 72 h after 2. SAH; Treatment: 24 and 72 h after 2. SAH	Statin attenuated SAH-induced neuron apoptosis inhibiting cJNK (p46/p55) activation and reducing caspase 9a expression
Duan et al. (28)	Rabbits	Double blood injection model	*n =* 24/group	20 mg/kg/day **Atorvastatin**	Oral	Daily for 3 days before and also at 22 h after SAH	Evaluation of statin effect on early brain injury, cerebral edema, and its association with Aquaporine 4	72 h after SAH	Amelioration of brain edema and early brain injury after SAH inhibiting AQP4 under atorvastatin treatment
Chen et al. (29)	Rabbits	Blood injection model	*n =* 12/group	5 mg/kg/day **Simvastatin**	Oral	Precondition for 1 week before SAH induction	Evaluation of Basilar artery diameter by cerebral angiography; Gene expression of vasoactive factors	Before 1. blood injection and 3 days after the second injection	Relieving cerebral vasospasm after SAH
Chen et al. (30)	Rabbits	Double blood injection model	*n =* 16/group	20 mg/kg/day **Atorvastatin**	Oral	3 days prior to SAH operation and 1x/day for 72 h post-SAH	Measurement of cross-sectional areas of BA; Evaluation of vasoactive factors and neuronal apoptosis.	72 h after SAH	Atorvastatin treatment may alleviate cerebral vasospasm and mediate structural and functional remodeling of vascular endothelial cells

### Patient Characteristics

A total of 293 patients with naSAH were included. 51 (17%) naSAH patients with simvastatin treatment (40 mg/d) were recruited for analysis, including 31 (61%) patients with pretreatment and 20 (39%) patients with post-treatment after SAH onset. In total 54 patients (18%) developed early hydrocephalus, 62 patients (21%) cerebral vasospasm, 53 patients (18%) had delayed cerebral ischemia (DCI) and 68 patients (23%) suffered from delayed infarction at discharge. Concerning Fisher bleeding pattern on CT-scan at admission, a total of 86 (29%) patients with a Fisher 3 bleeding pattern were included.

Patients under simvastatin treatment were affected by a significantly lower risk of Fisher 3 bleeding pattern on initial CT-scan (*P* < 0.05; OR 13.5), early hydrocephalus (*P* < 0.05; OR 2.5); CVS (*p* < 0.01; OR 3.7), DI (*p* < 0.05; OR 2.6), and DCI (*p* < 0.05; OR 3.0). Additionally, we differentiate between CVS-related (22/68, 32%) and non-CVS-related (46/68, 68%) DI describing a trend toward lower risk of CVS-related DI (1/22, 5%) in patients with simvastatin treatment (Table 2A).

**Table 2A:** Clinical complications and outcome in NASAH patients in dependence on statin treatment between 1999 and 2018.

**Complications after NASAH**	**NASAH**	**No Statin treatment**	**Statin treatment**	^*^p-value ** Odd Ratio (95%CI)**	**Statin** ** pre-treatment**	**Statin** ** post-SAH-treatment**	^*^p-value ** Odd Ratio (95%CI)**
**Number of pat**.	293	242 (83%)	51 (17%)	–	31 (61%)	20 (39%)	–
**Mean age in years**	54 ± 11	51 ± 15	53 ± 12	NS *p =* 0.86	54 ± 11	52 ± 8	NS *p =* 0.91
**Perimesencephalic naSAH**	169 (58%)	146 (86%)	23 (14%)	NS *p =* 0.06	12 (52%)	11 (48%)	NS *p =* 0.39
**Non-perimesencephalic naSAH**	124 (42%)	96 (77%)	28 (23%)	NS *p =* 0.06	19 (68%)	9 (32%)	NS *p =* 0.39
***Fisher 3 bleeding pattern***	***86 (29%)***	***61 (71%)***	***25 (29%)***	***p<0.05** ** OR 13.5 (6.5–27)***	***17 (68%)***	***8 (32%)***	***NS** ** P=0.39***
**Cerebral vasospasm (CVS)**	**62 (21%)**	**58 (94%)**	**4 (6%)**	***p<*** **0.05** **OR 3.7 (1.3–9.9)**	3 (75%)	1 (25%)	NS *p >* 0.999
**Delayed infarction (DI)**	**68 (23%)**	**62 (91%)**	**6 (9%)**	***p<*** **0.05** **OR 2.6 (1.1–6.0)**	4 (67%)	2 (33%)	NS *p >* 0.999
*CVS-related DI*	22 (32%)	21 (95%)	1 (5%)	NS *p =* 0.66	1 (100%)	0 (0%)	NS *p =* 0.39
*non-CVS-related DI*	46 (68%)	41 (89%)	5 (11%)	NS *p =* 0.66	3 (60%)	2 (100%)	NS *p =* 0.37
**Delayed cerebral ischemia (DCI)**	**53 (18%)**	**49 (92%)**	**4 (8%)**	***p<*** **0.05** **OR 3.0 (1.1–8.0)**	3 (75%)	1 (25%)	NS *p >* 0.999
**Early hydrocephalus**	**54 (18%)**	**38 (70%)**	**16 (30%)**	***p<*** **0.05 OR 2.5 (1.3–5)**	**7 (44%)**	**9 (56%)**	NS *p =* 0.13
**Shunt implantation**	31 (11%)	27 (87%)	4 (13%)	NS *p =* 62	2 (50%)	2 (50%)	NS *p =* 0.64
**Favorable outcome mRS 0–2**	**240 (82%)**	**193 (80%)**	**47 (20%)**	***p<*** **0.05** **OR 3.0 (1.1–8.0)**	27 (57%)	20 (43%)	NS *p =* 0.15

*Favorable outcome 6 months after SAH: mRS ≤ 2 points; Unfavorable outcome: mRS >2 points. Data are shown in n (%); Fisher exact test:*

* *p < 0.05 is significant. Odd ratio (OR) data with 95% confidence interval. Bold values indicate significant parameter*.

Furthermore, our results indicate a significant association between simvastatin treatment and favorable neurological outcome (*p* < 0.05; OR 3.0). A relevant statistical difference between simvastatin-pretreatment and –post-SAH-treatment was not detected in this small cohort (Table 2A).

Due to the pathomechanism of statin effect as a treatment option after naSAH based on recent clinical studies, we performed Table 2B analyzing SAH followed complications and outcome in case of patients with statin post-SAH treatment compared to untreated group. Even in this small patient group with statin post-SAH treatment (*n* = 20), we detected a significant higher chance for favorable outcome (*p* < 0.05; OR 0.05) in these patients and a trend toward lower risk for development of cerebral vasospasm (*p* = 0.05) (Table 2B). Nevertheless, because of the small number of patients (*n* = 20) these statistical results should be treated with caution.

**Table 2B:** Clinical complications and outcome in NASAH patients in dependence on statin post-SAH-treatment between 1999 and 2018.

**Complications after NASAH**	**NASAH**	**No Statin** ** treatment**	**Statin** ** post-SAH-treatment**	^*^p-value Odd Ratio (95%CI)
**Number of pat**.	293	242 (83%)	20 (7%)	–
**Mean age in years**	54 ± 11	51 ± 15	52 ± 8	NS *p =* 0.94
**Perimesencephalic naSAH**	169 (58%)	146 (60%)	11 (55%)	NS *P =* 64
**Non-perimesencephalic naSAH**	124 (42%)	96 (40%)	9 (45%)	NS *P =* 0.64
***Fisher 3 bleeding pattern***	86 (29%)	61 (25%)	8 (40%)	NS *P =* 0.19
**Cerebral vasospasm (CVS)**	***62 (21%)***	***58 (24%)***	***1 (5%)***	***NS P=0.05***
**Delayed infarction (DI)**	68 (23%)	62 (26%)	2 (10%)	NS *P =* 0.17
*CVS-related DI*	22 (32%)	21 (34%)	0 (0%)	NS *p >* 0.99
*non-CVS-related DI*	46 (68%)	41 (66%)	2 (100%)	NS *p >* 0.99
**Delayed cerebral ischemia (DCI)**	53 (18%)	49 (20%)	1 (5%)	NS *p =* 0.14
**Early hydrocephalus**	54 (18%)	38 (16%)	9 (45%)	NS *p >* 0.99
**Shunt implantation**	31 (11%)	27 (11%)	2 (10%)	NS *p >* 0.99
**Favorable outcome** **mRS 0–2**	**240 (82%)**	**193 (80%)**	**20 (100%)**	***p<*** **0.05** **OR 0.05 (0–0.7)**

* *Favorable outcome 6 months after SAH: mRS ≤ 2 points; Unfavorable outcome: mRS >2 points. Data are shown in n (%); Fisher exact test:*

*p < 0.05 is significant. Odd ratio (OR) data with 95% confidence interval. Bold values indicate significant parameter*.

Using a multivariate analysis to detect significant patient characteristics associated with favorable outcome, we recognized female gender (55%; *p* < 0.001; 4.9), Hunt & Hess ≤ III at admission (*p* < 0.002; 4), no anticoagulation and antiplatelet drug treatment (*p* < 0.0001; 0.16), and statin treatment (*p* < 0.0001; 24.2) as main factors improving the clinical outcome of naSAH patients (Table 3).

**Table 3:** Clinical outcome in association with patient characteristics in NASAH patients admitted to the University Hospital (City) between 1999 and 2018.

**Pat. characteristics**	**NASAH**	**Favorable outcome**	**Unfavorable outcome**	Multivariate analysis ^*^p-value; OR (95% CI)
**Number of patients**	**293**	**240** (82%)	**53** (18%)	–
**Age** **≤** **50 years**	173 (59%)	120 (69%)	53 (31%)	NS
**Female**	**162 (55%)**	**116 (72%)**	**46 (28%)**	***p<*** **0.001; 4.9 (1.9–12.3)**
**Hunt&Hess** **≤** **III**	**148 (51%)**	**103 (70%)**	**45 (30%)**	***p<*** **0.002; 4 (1.7–9.5)**
**Fisher 3 blood pattern**	86 (29%)	40 (47%)	46 (53%)	NS
**Hypertension**	134 (46%)	84 (63%)	50 (37%)	NS
**Statin treatment**	**51** (17%)	**47** (92%)	**4** (8%)	***p<*** **0.0001; 24.2 (5.9–99.2)**
***Statin post-SAH treatment***	**20 (7%)**	**20 (100%)**	**0 (0%)**	***p<*** **0.05; 0.03 (0–0.76)**
**Active smoking**	88 (30%)	42 (48%)	46 (52%)	NS
**Early hydrocephalus**	54 (18%)	19 (35%)	35 (65%)	NS
**Anticoagulation and antiplatelet drugs**	**73 (25%)**	**29 (12%)**	**44 (83%)**	***p<*** **0.0001; 0.16 (0.06–0.46)**
**Positive family history**	23 (8%)	10 (43%)	13 (57%)	NS

*Favorable outcome 6 months after SAH: mRS ≤ 2 points; Unfavorable outcome: mRS >2 points. Data are shown in n (%); Multivariate analysis:*

* *p < 0.05 is significant. Odd ratio (OR) data with 95% confidence interval. Bold values indicate significant parameter*.

## Discussion

Creating a scientific basis as a background for the evaluation of data of included naSAH patients, we firstly attempted to collect all experimental studies focused on statin effect in animals using the blood injection model, according to the pathophysiology of naSAH.

We detected 13 experimental studies differing on study design factors including animal species, drug dose and application route, time of drug administration, employed tests, and time of examination. Concerning different conclusions, six studies described an attenuation of cerebral vasospasm after SAH onset, one study could prove ameliorated neurological deficit after SAH, another study dealt with brain edema after SAH, being attenuated under statin treatment and a single publication determined a decreased rate of cognitive dysfunction. Two further included studies focused on the mechanism of statin effect. Taking all studies paying attention to the statin effect on cerebral vasospasm under consideration, we could not unify the results to be analyzed as a metanalysis, not only because of the different study setup, but also because of the different evaluations of severity of vasospasm. Except for two included experimental research studies, performing cerebral angiography in experimental animals after SAH induction as the most precise established method to detect vasospasm, other studies choose to measure cross-sectional areas of vessels histologically, which does not allow an accurate assessment of vessel diameters.

Considering all mentioned factors, independent of all scientific research work invested in these publications, there is no possibility to unify the resulting statements to enable a statistically crucial analysis as a background for further clinical studies.

However, despite all of these discrepancies, most studies concentrating on statin effect following complications after SAH used blood injection models to induce the bleeding, which does mimic non-aneurysmal SAH rather than aneurysmal SAH. All of these experimental studies underlined the positive effect of statin treatment after SAH induction from different points of view. Investigating numerous clinical studies on this aspect in aneurysmal SAH patients and failing to confirm experimental findings, a retrospective analysis to determine the possible reasons of these discrepancies was not in the focus of interest. Even multiple published meta-analyses produced inconsistent results (8–15).

However, two further large-scale multicenter Phase III trials, STASH (16) and HDS-SAH (17), resulted in disappointing findings. The STASH study could not confirm an improved outcome in aneurysmal SAH patients. In addition, authors of the HDS-SAH study denied any benefits of treatment with high doses of simvastatin (80 mg) vs. treatment with low doses (40 mg), as described previously. Nevertheless, both studies had limitations. In the STASH study the evaluation of vasospasm was not described in detail, and in the HDS-SAH study no control group was used. These limiting factors make it difficult to draw evidence-based conclusions (29).

Therefore, the effect of statins on vasospasm after SAH remained unclear; positive effects may have been neutralized through an adverse pathway. However, recognizing this fact and regarding the pathophysiology of the experimental SAH model being used in most of the studies done in this field, we created a translational insight detecting naSAH patients to assess the statin effect.

Previous studies observed a significant increase in the number of patients with NASAH. Non-aneurysmal SAH patients have significantly less DCI and a higher chance for excellent outcomes compared to aneurysmal SAH, but patients with PM and NPM-SAH also developed DCI. Especially patients with an NPM-SAH have, according to the bleeding pattern, an increased risk for DCI and for a worse neurological outcome (32). Therefore, patients with a NASAH should be monitored for new therapeutic options concerning SAH followed complications to increase the chance for a favorable outcome.

This is the first study presenting the statin effect in patients with naSAH in accordance to the pathophysiological background of statin treatment attenuating SAH following complications in animals. We could detect a significantly lower risk for CVS, DCI, and DI in naSAH patients under statin treatment. Additionally, a significant association between statin treatment and favorable outcome 6 months after naSAH onset could be confirmed. According to previous studies, patients with NASAH and a Fisher Grade 3 bleeding pattern had a significantly higher risk for an unfavorable outcome and death (32). However, a multivariate analysis of recent data described the bleeding pattern as a not-significant risk factor for unfavorable outcome.

Although there is a red thread through the data, from bench to bedside, underlining the effect of statin treatment in naSAH patients, there are some limitations in this study, which have to be under consideration. At first, the small number of detected patients did not allow differentiations to be made between patients under different drugs and doses of statin. In addition, an analysis of statin effect as a pre-treatment vs. post-SAH-treatment could not be done. This aspect should be considered as a limiting factor, because detecting 20 patients suffering from naSAH with statin post-treatment could not be sufficiently powered by statistical analysis to prove the therapeutic effect of statins in the case of the clinical course of naSAH patients. Nevertheless, a separated analysis of the small patient group with statin treatment after SAH detected a tenuous significantly higher chance for favorable outcome and a trend toward lower risk for cerebral vasospasm after non-aneurysmal SAH (Table 2B).

However, recent data demonstrate a positive trend in case of statin-effect in naSAH patients according to the known important role of statins in vasculogenesis and vascular regeneration (31).

Furthermore, dividing non-aneurysmal SAH into perimesencepahlic (PM-) SAH, where the blood is strictly around the midbrain or brainstem, and non-perimesencephalic (NPM-) SAH, where the blood extends into the adjacent cistern, it is well-known that NPM-SAH have a different illness course with higher risk of CVS, DCI and poor outcome compared to PM-SAH (32). Concerning this aspect, because of the small number of included patients we could not differ between PM-SAH and NPM-SAH in our multivariate analysis. All these aspects minimize the impact of these results.

Therefore, a combination of pre-/post-treatment with statins may play a role, but a causal relationship cannot, thus far, be proven. Data from other centers are necessary to confirm our findings; however, for this rare condition, the cohort may be considered large.

However, contrary to the most studies in aneurysmal SAH patients, there is a significant association between statin treatment and the occurrence of cerebral vasospasm, delayed infarction, and delayed cerebral ischemia in the case of non-aneurysmal SAH. To analyze propriety and possible application of our results in daily clinical management of naSAH patients, further prospective clinical trials are needed.

Furthermore, by failure of multiple big size clinical studies despite successful experimental data, a translational flashback could ease the way to recognize possible influencing factors. Therefore, unified animal experiments and translational clinical trials should be considered to create the basis for developing new therapeutic schemes, successfully.

Based on this translational study, the injection models mimic non-aneurysmal SAH rather than aneurysmal SAH, and the model might not be suitable to study aneurysmal SAH.

## Conclusion

Collecting all experimental studies focused on statin effect in animals using the blood injection model, we firstly detected the accordance of this SAH model with the pathophysiology of naSAH.

Then, we presented the statin effect in patients with naSAH in accordance with the pathophysiological background of statin treatment attenuating SAH following complications in animals. We could detect a significant lower risk for CVS, DCI, and DI in naSAH patients being under statin treatment. Additionally, a significant association between statin treatment and favorable outcome 6 months after naSAH onset could be confirmed. Nevertheless, unified animal experiments should be considered to create the basis for developing new therapeutic schemes.

## Data Availability Statement

The original contributions presented in the study are included in the article/supplementary material, further inquiries can be directed to the corresponding author/s.

## Author Contributions

SK and JK wrote the main manuscript text and SK prepared Figure 1 and Tables 1–3. All authors reviewed the manuscript.

## Conflict of Interest

The authors declare that the research was conducted in the absence of any commercial or financial relationships that could be construed as a potential conflict of interest.
